# Supplementary material for: Complexome profiling of the Chlamydomonas psb28 mutant reveals TEF5 as an early PSII assembly factor
Source: Plant Cell. 2025 Mar 17;37(6):koaf055. doi: 10.1093/plcell/koaf055 (PMC12164590; doi:10.1093/plcell/koaf055)
Supplement: koaf055_Supplementary_Data [file koaf055_supplementary_data.zip › psb28_interactive.zip!/plots/intensity/qualityPlots/peptides/_Cre06.g279400.t1.2.html]

### Cre06.g279400.t1.2

Trivial name:   
SeqCoverage [median]: 13.80  
Mass: 64.21 kDa  
  
 

| Sequence | MissedCleavage | Mass [Da] | wtIntensity | muIntensity | Score |
| --- | --- | --- | --- | --- | --- |
| AHPEVAENPFYVTGESYAGHYVPAVSSAIYR | 0 | 3380.61 | 1647.82 | 0.00 | 49.79 |
| ELGTLSFVR | 0 | 1020.56 | 845.62 | 14045.78 | 101.54 |
| ILGANPDINVYDITK | 0 | 1644.87 | 1520.00 | 5576.02 | 164.04 |
| ILGANPDINVYDITKK | 1 | 1772.97 | 2705.63 | 10612.16 | 65.23 |
| VGRDMLDFLYEFYR | 1 | 1822.87 | 720.67 | 0.00 | 51.55 |
|  |  |  | 7439.7 | 30234.0 |  |
